# iTRAQ-Based Analysis of Proteins Co-Regulated by Brassinosteroids and Gibberellins in Rice Embryos during Seed Germination

**DOI:** 10.3390/ijms19113460

**Published:** 2018-11-04

**Authors:** Qian-Feng Li, Jin-Dong Wang, Min Xiong, Ke Wei, Peng Zhou, Li-Chun Huang, Chang-Quan Zhang, Xiao-Lei Fan, Qiao-Quan Liu

**Affiliations:** 1 Key Laboratory of Crop Genetics and Physiology of Jiangsu Province/Key Laboratory of Plant Functional Genomics of the Ministry of Education/Jiangsu Key Laboratory of Crop Genomics and Molecular Breeding, College of Agriculture, Yangzhou University, Yangzhou 225009, China; qfli@yzu.edu.cn (Q.-F.L.); wangjd1012@163.com (J.-D.W.); xiongmin199401@163.com (M.X.); weike1015@sina.com (K.W.); jayzhou785@outlook.com (P.Z.); huanglichun@outlook.com (L.-C.H.); cqzhang@yzu.edu.cn (C.-Q.Z.); xlfan@yzu.edu.cn (X.-L.F.); 2 Co-Innovation Center for Modern Production Technology of Grain Crops of Jiangsu Province/Joint International Research Laboratory of Agriculture and Agri-Product Safety of the Ministry of Education, Yangzhou University, Yangzhou 225009, China

**Keywords:** brassinosteroid, gibberellin, seed germination, iTRAQ, *Oryza sativa*

## Abstract

Seed germination, a pivotal process in higher plants, is precisely regulated by various external and internal stimuli, including brassinosteroid (BR) and gibberellin (GA) phytohormones. The molecular mechanisms of crosstalk between BRs and GAs in regulating plant growth are well established. However, whether BRs interact with GAs to coordinate seed germination remains unknown, as do their common downstream targets. In the present study, 45 differentially expressed proteins responding to both BR and GA deficiency were identified using isobaric tags for relative and absolute quantification (iTRAQ) proteomic analysis during seed germination. The results indicate that crosstalk between BRs and GAs participates in seed germination, at least in part, by modulating the same set of responsive proteins. Moreover, most targets exhibited concordant changes in response to BR and GA deficiency, and gene ontology (GO) indicated that most possess catalytic activity and are involved in various metabolic processes. Search Tool for the Retrieval of Interacting Genes/Proteins (STRING) analysis was used to construct a regulatory network of downstream proteins mediating BR- and GA-regulated seed germination. The mutation of *GRP*, one representative target, notably suppressed seed germination. Our findings not only provide critical clues for validating BR–GA crosstalk during rice seed germination, but also help to optimise molecular regulatory networks.

## 1. Introduction

As sessile organisms, plants need to modulate their growth and development constantly to adapt to the changing environment. A variety of environmental cues, including photoperiod, temperature, and biotic and abiotic stress, are sensed by plants and acted upon by intrinsic hormonal pathways to help determine whether it is appropriate to grow or not [1,2]. At present, the known plant hormone family includes at least nine classes, including auxins, gibberellins (GAs), abscisic acid (ABA), cytokinin (CKs), ethylene, brassinosteroids (BRs), jasmonate (JA), salicylic acid (SA), and strigolactones [3,4]. Although the major functions of each hormone class have been clearly revealed by a series of physiological and genetic studies, the final output that affects plant growth and development is determined by a complex regulatory network mediated by hormonal interactions, and the importance of hormone–hormone interplay is becoming more obvious [5,6,7,8]. 

Among phytohormones, BRs and GAs are two major growth-promoting hormones with extensive overlapping functions in various plant growth and developmental processes including cell elongation, seed germination, flowering, male fertility and senescence [9]. Interestingly, a great deal of evidence suggests that crosstalk between BRs and GAs regulates plant growth and development. For instance, physiological studies indicate that co-application of BRs and GAs results in a synergistic increase in hypocotyl elongation of light-grown plants [10]. Knock-down of the expression of *SPINDLY* (*SPY*), a negative regulator of GA signalling, enhances BR biosynthesis and enlarges lamina joint bending, which represents a BR-related phenotype [11]. Impressive progress has been made in our understanding of the actions of BRs and GAs, especially in their signalling mechanisms and interactions. Two major molecular mechanisms have been established to illustrate the crosstalk between BR and GA pathways. One mechanism, the so-called ‘signalling model’, depends on the physical interaction between BRASSINAZOLE-RESISTANT 1 (BZR1)/BRI1-EMSSUPPRESSOR 1 (BES1) transcription factors and DELLA proteins [12,13,14]. BZR1 and BES1 play critical roles in the BR-signalling pathway by directly regulating a large number of target genes in the *Arabidopsis* genome [15,16]. The DELLA protein is the master negative regulator of the GA pathway, and undergoes 26S proteasome pathway-mediated degradation in response to bioactive GA3, resulting in the release of plant growth suppression [17,18]. Thus, DELLA directly interacts with BZR1/BES1 and thereby attenuates their stability and activity, and ultimately restrains plant cell elongation [12,13,14]. In the other mechanism, the so-called ‘synthesis model’, BZR1/BES1 binds directly to the promoters of several GA metabolic genes, including *GA20ox*, *GA3ox* and *GA2ox*, and thereby modulates their expression and promotes cell elongation in both *Arabidopsis* and rice [19,20]. However, how these two different models are integrated together to help adapt to changing internal and external stimuli remains elusive. Moreover, the mechanisms could act differently in different organs or tissues, and whether the two mechanisms are involved in the regulation of other cellular or developmental programs in addition to cell elongation remains unknown. Recently, a mathematical model was also established to predict the influence of crosstalk between BRs and GAs on plant growth and development [21]. Furthermore, there may also be other mechanisms involved in the crosstalk between BRs and GAs. For example, the chromatin remodelling factor PICKLE functions as a critical node that integrates not only BR and GA pathways, but also light signalling to regulate plant growth and development epigenetically [22]. JUNGBRUNNEN1 (JUB1), a NAC *(NAM, ATAF1/2, CUC1/2)* transcription factor, regulates the metabolism and signalling of BRs and GAs in *Arabidopsis* [23] and tomato [24]. Recently, Tang et al. (2018) reported that OsmiR396d also participates in the BR–GA interaction module, thereby coordinating plant architecture in rice [25]. More recently, a new BR-responsive miRNA-target module was identified, that is BR could promote the expression of *OsGAMYBL2* by suppressing the level of OsmiR159d. In addition, both OsGSK2, a key negative player in BR signaling, and SLR1, a rice DELLA protein, could directly interact with OsGAMYBL2 to modulate its stability and activity, respectively, thus to coordinate the regulation of BR and GA in plant growth and development [26]. Nevertheless, it remains obscure whether these proposed models and characterised integrators are also suitable for other developmental process, such as seed germination. Indeed, recent findings in maize suggest that BRs and GAs do not interact via a single inclusive pathway [27]. Rather, BR and GA signal transduction and downstream responses are affected by the developmental context.

Seed germination is a pivotal process in the life cycle of higher plants, which is regulated by a series of external and internal clues, including plant hormones [28,29]. It has long been acknowledged that BRs and GAs co-regulate seed germination [30,31,32]. A previous RNA-seq analysis of rice embryos disclosed the phytohormone interaction network during seed germination, and six of the top 10 hub genes belong to GA, BR and ABA pathways [33], highlighting their key roles in rice seed germination. However, the underlying molecular mechanism of their interaction is still obscure. Moreover, rice is not only a monocotyledonous model plant, but also an important food crop as the staple food for more than half of the world’s population. Furthermore, although it is noteworthy that rice contains a relatively small embryo and a dominant endosperm, the rice embryo contains most of the genetic information and plays a decisive role during rice seed germination [34]. Thus, studying the interplay between BRs and GAs in the rice embryo during seed germination is important.

Proteomic analysis is a powerful tool for identifying changes in proteins in response to various internal and external stimuli [35]. Following on from the traditional two-dimensional gel electrophoresis (2-DE) technique, isobaric tags for relative and absolute quantification (iTRAQ)-based proteomic analysis is a new quantitative method that can quantitatively analyse protein abundance in eight samples simultaneously with high confidence and repeatability [36]. It has been used to identify multiple biological processes in various plant species including *Arabidopsis* [37], rice [38], wheat [39], maize [40]. However, this powerful method has not been applied to investigate how BRs and GAs coregulate rice seed germination, and information regarding the interplay between BRs and GAs during seed germination remains limited. Therefore, in the present study, the iTRAQ proteomic approach was performed to identify the common responsive proteins of BRs and GAs in rice embryos during the most important stage for seed germination (Phase II), thus to help reveal the underlying molecular mechanism of their crosstalk in coordinating rice seed germination.

## 2. Results

### 2.1. Generation of the Nip (sd1) Near-Isogenic Line (NIL)

**Figure 1:**
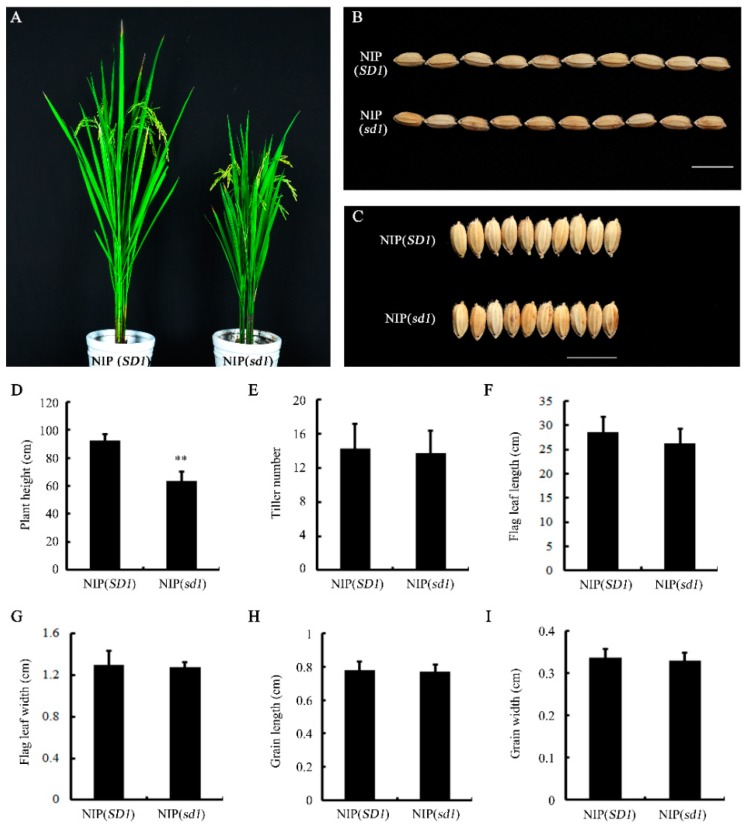
Phenotype of near-isogenic line (NIL) NIP (*sd1*). (**A**) Gross morphology of NIP (*SD1*) and NIP (*sd1*) NILs grown in the paddy field. (**B**) Seed length of (top) NIP (*SD1*) and (bottom) NIP (*sd1*) NILs. (**C**) Seed width of (top) NIP (*SD1*) and (bottom) NIP (*sd1*) NILs. Scale bar = 1 cm. (**D**) Plant height, (**E**) tiller number, (**F**) flag leaf length, (**G**) flag leaf width, (**H**) grain length, and (**I**) grain width of NIP (*SD1*) and NIP (*sd1*) NILs.

In our previous work, we generated a series of chromosome segment substitutional lines (CSSLs) using the *japonica* variety Nip as the recipient parent and *indica* variety 9311 as the donor parent. Among the CSSL lines, the line CSSL120 exhibited a notable semi-dwarf phenotype. After several rounds of backcrossing with Nipponbare and map-based cloning, we finally confirmed that it is the *SD1* locus, encoding the GA biosynthesis enzyme GA 20 oxidase (GA20ox), that causes the semi-dwarf phenotype. Alignment of the *SD1* coding sequence (CDS) in Nipponbare and 9311 indicated the existence of four single-nucleotide polymorphisms (SNPs), among which one SNP (654) did not change an amino acid, two SNPs (299 and 1019) resulted in an amino acid substitution (Glu to Gly and Gln to Arg, respectively), and the remaining SNP (1026) changed a Tyr residue to a stop codon (Figure S1). Thus, the *SD1* allele in 9311 encodes a mutated and defective form of GA20ox, resulting in the GA-deficient endogenous condition. One progeny of the backcross line between CSSL 120 and Nip was genotyped and selected as the NIL of *SD1* in the Nip background, designated as Nip (*sd1*) or *sd1*, and used in subsequent seed germination and proteomic analysis (Figure S2). In general, *sd1* exhibited a semi-dwarf phenotype in terms of plant height (Figure 1A,D), but its other agronomic traits, including grain size, tiller number, length and width of flag leaf, were similar to that of the Nip (*SD1*) control (Figure 1B,C,E–I). Therefore, seeds of Nip (*sd1*) were ideal materials for investigating how GA affects rice germination.

### 2.2. Seed Germination Assays with Either Brassinosteroid (BR) or Gibberellin (GA) Biosynthesis Blocked

**Figure 2:**
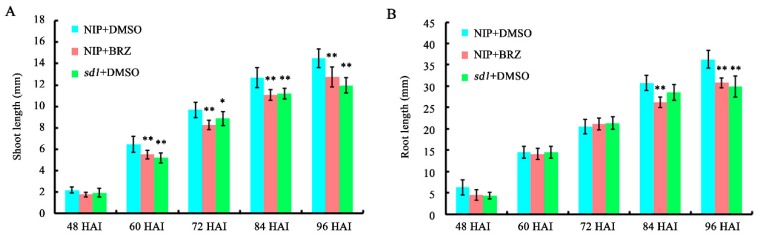
Both brassinosteroid (BR) and gibberellin (GA) deficiency suppress rice shoot and root elongation during seed germination. (**A**) Shoot length of germinated seeds. (**B**) Root length of germinated seeds. Asterisks indicate significant differences between NIP/brassinazole (BRZ) or sd1/dimethyl sulphoxide (DMSO) and NIP/DMSO by Student’s *t* tests (* *p* < 0.05, ** *p* < 0.01).

Shoot and root length of germinated seeds were measured every 12 h, from 48 to 96 hours after imbibition (HAI). The results indicate that both BRZ treatment and *SD1* mutation led to a significant reduction in shoot length during most tested stages (Figure 2A). Regarding root length, a significant change in BR-deficient seeds was observed after 84 HAI, but a change was only evident after 96 HAI for GA-deficient seeds (Figure 2B). These results suggest that both BR and GA play essential roles during rice seed germination. 

### 2.3. Expression Changes in Embryo Proteins in Response to BR or GA Deficiency during Seed Germination

**Figure 3:**
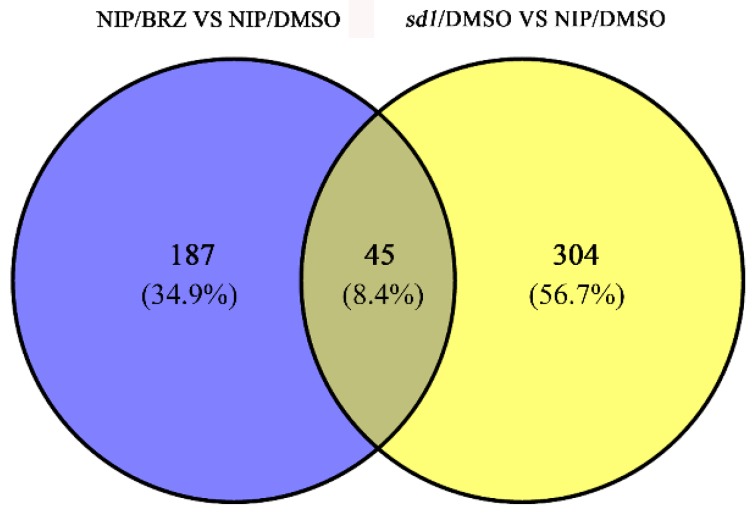
Venn diagram illustrating the number of common responsive proteins identified from embryos of germinated seeds in both BRZ-treated NIP and the *sd1* mutant.

Since proper translation in early stage of germination, especially phase II, is a prerequisite for germination, the embryos of germinated seeds after 36 HAI were used for proteomic analysis. Here, the non-gel-based iTRAQ technique was applied to identify common proteins in the response to both BR and GA deficiency. The embryos were isolated for proteomic assays, and total proteomes from three different origins, Nip/BRZ, Nip/DMSO and *sd1*/DMSO, were labelled by iTRAQ and quantified to identify differentially expressed proteins. Specifically, protein abundance was compared between Nip/BRZ and Nip/DMSO, and between *sd1*/DMSO and Nip/DMSO, respectively. Based on MS/MS analysis and comparison of the results, 45 proteins were identified as common targets in responses to both BR and GA deficiency (Figure 3). Among these, expression of 29 proteins was changed consistently in response to both BR and GA deficiency, of which 15 were down-regulated (Table 1) and 14 were up-regulated (Table 2). Among the 29 proteins, five (glutelin B2, prolamin, glycine-rich protein, pectin esterase-like protein, and acyl-desaturase) displayed distinct expression changes (>1.5-fold) in response to both BRZ and *sd1* mutation (Figure S3). Meanwhile, 16 proteins exhibited differences in expression pattern in BRZ-treated Nip and mock-treated *sd1* rice (Table 3). Thus, the 45 common differentially expressed proteins responsive to both BR and GA deficiency represented candidates for investigation of crosstalk in the coordination of rice seed germination.

### 2.4. Bioinformaticf Analysis of Common Responsive Proteins Co-Regulated by BR and GA

**Figure 4:**
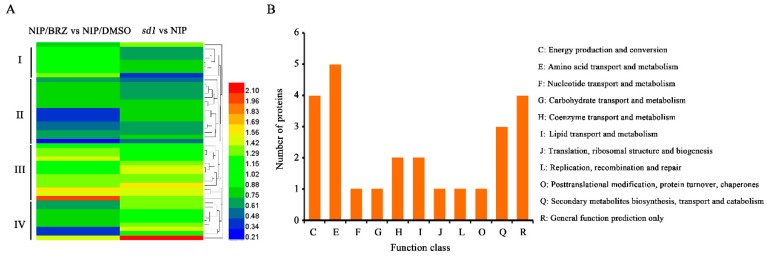
Hierarchical cluster analysis and Clusters of Orthologous Groups (COG) classification of differentially abundant proteins in the embryos of BRZ-treated NIP and the *sd1* mutant. (**A**) Hierarchical cluster analysis of common proteins in response to both BRZ treatment and *OsGA20ox2* mutation. (**B**) COG classification of all common proteins.

To gain knowledge of differentially expressed proteins common to both BR and GA in the regulation of seed germination, a number of bioinformatic analyses were performed, including hierarchical clustering, which is widely applied for grouping proteins exhibiting similar expression changes in omics studies. Accordingly, the 45 common proteins were classified into clusters I to IV (Figure 4A). Most proteins in cluster II and III shared concordant expression patterns; protein abundance in cluster II was markedly down-regulated and that in cluster III was up-regulated in both BRZ-treated NIP and the mock-treated *sd1* mutant, suggesting that BR and GA deficiency resulted in similar expression changes for many common targets. Using the Clusters of Orthologous Groups (COG) database, the 45 proteins were further divided into different functional categories (Figure 4B), among which the ‘Amino acid transport and metabolism’ category included five proteins, and the ‘Energy production and conversion’ and ‘General function prediction only’ groups each contained four proteins.

**Figure 5:**
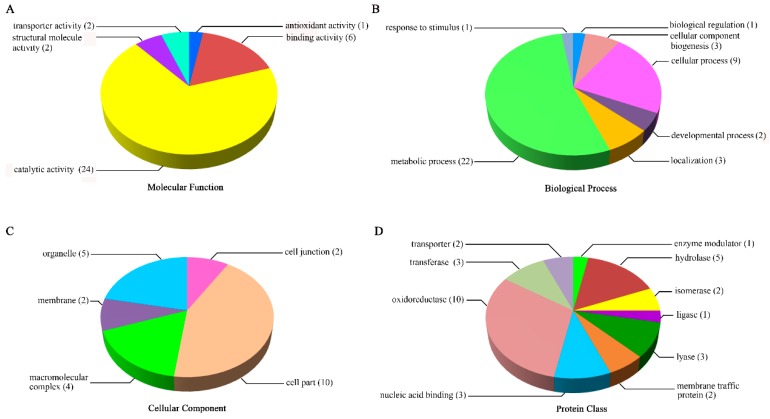
Functional analyses of proteins responsive to both BR and GA deficiency among different gene ontology (GO) categories in embryos of germinated seeds. Common responsive proteins are divided into molecular function (**A**), biological process (**B**), cellular component (**C**) and protein class (**D**).

The results of molecular function analysis showed that all test proteins could be classified into five functional groups (catalytic activity, binding activity, structural molecule activity, transporter activity, and antioxidant activity). Among these, almost 70% of total proteins belonged to the ‘catalytic activity’ group, and the ‘binding activity’ group was the next most enriched (Figure 5A). Based on GO analysis of biological processes, more than half of test proteins were classified into the ‘metabolic process’ group. The second largest group was the ‘cellular process’ group, containing more than 25% of total proteins. Additionally, a few proteins belonged to five other groups (localisation, cellular component biogenesis, developmental process, biological regulation, and response to stimulus; Figure 5B). When the proteins were classified according to cellular component, the ‘cell part’ group contained the largest number of proteins, followed by the ‘organelle’ and ‘macromolecular complex’ groups (Figure 5C). Finally, the results of GO analysis of the protein class showed that these proteins could be grouped into 10 subfamilies, among which oxidoreductases accounted for the largest proportion (31.2%), followed by hydrolase (15.6%), transferase (9.4%), nucleic acid binding (9.4%), and lyase (9.4%; Figure 5D).

**Figure 6:**
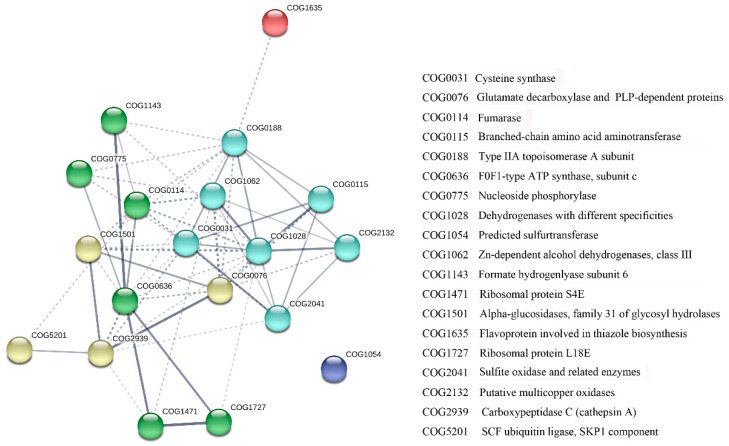
Search Tool for the Retrieval of Interacting Genes/Proteins (STRING) construction of a potential protein-protein interaction network responsive to both BR and GA deficiency during seed germination. The network, made with a medium confidence cutoff (0.4) using the k-means clustering method, includes five clusters presented as different colours. Line thickness indicates the strength of data support.

Dissecting the complex network of protein-protein interactions (PPI) could facilitate a better understanding of the molecular mechanism of the crosstalk between BR and GA in seed germination. Totally 19 proteins were included in the constructed network (Figure 6), which was made with a medium confidence cutoff (0.4) using the k-means clustering method. Interestingly, 17 proteins (89.5%) had a high degree of connectivity (more than 2 connections) and were centered on three main node (protein) clusters. Proteins with the highest connectivity are mainly involved in the metabolic process, such as alcohol dehydrogenase, ATP synthase and Type IIA topoisomerase, suggesting the metabolic process is the critical regulation node of seed germination by BR and GA.

### 2.5. Validation of Several Representative Genes by qRT-PCR

**Figure 7:**
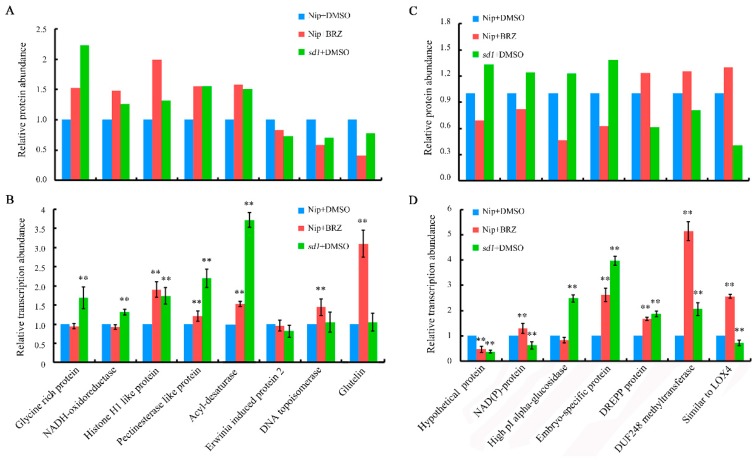
Validation of proteomic data by quantitative real-time polymerase chain reaction (qRT-PCR). (**A**) Eight selected common responsive proteins displaying consistent changes in protein abundance in response to both BR and GA deficiency. (**B**) Changes in transcription for the eight selected genes. (**C**) Seven selected common responsive proteins displaying opposite changes in protein abundance in response to BR and GA deficiency. (**D**) Changes in transcription of the seven selected genes. Ubiquitin-Conjugating Enzyme (UBC) was used as an internal control. Asterisks indicate significant differences according to Student’s *t*-tests (** *p* < 0.01).

To further validate and complement our proteomic results, qRT-PCR analysis was performed to examine the transcriptions of 15 selected proteins co-regulated by both BR and GA. The selected proteins could be further classified into two different groups; group one displaying consistent protein pattern changes, and group two displaying opposite protein pattern changes (Figure 7A,B). In group one, the abundance of five proteins was elevated in response to both BR and GA deficiency, while three were down-regulated. In general, changes in transcript levels and protein abundance among targets were more consistent in the *sd1* mutant than in the BRZ-treated group. Interestingly, the transcription and translation of three targets, histone H1-like protein, pectin esterase-like protein, and acyl-desaturase, were all induced by both BR and GA deficiency (Figure 7B). In group two, although changes in protein abundance were opposite for all seven selected targets in the *sd1* mutant and BRZ-treated Nip, changes in transcription of some targets were consistent. For example, transcription of embryo-specific protein, DREPP protein, and DUF248 methyltransferase was significantly increased, while that of a hypothetical protein was notably decreased in both rice materials (Figure 7D). Furthermore, high isoelectric point (pI) alpha-glucosidase and acyl desaturase displayed consistent changes in transcription and protein abundance (Figure 7C,D).

### 2.6. Generation and Germination Analysis of grp Mutant 

**Figure 8:**
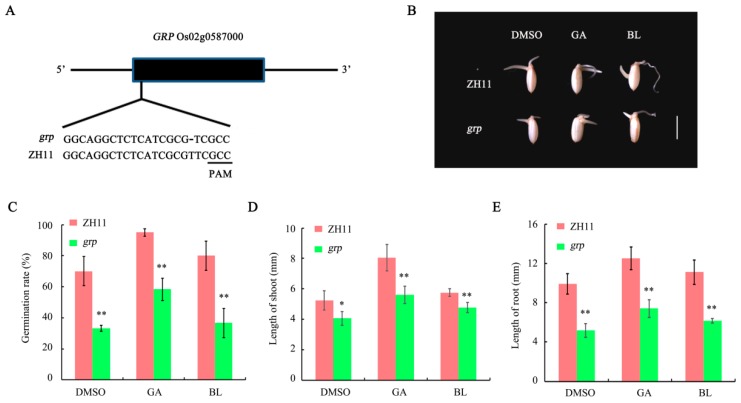
Germination analysis of *grp* mutant. (**A**) Schematic diagram of the target in *GRP* gene. PAM, the protospacer adjacent motif. (**B**) Rice seeds were dehulled and imbibed in Milli-Q water supplemented with GA, BL or DMSO solution in darkness for 84 h. The scale bar indicates 0.5 cm. (**C**) Rice seed germination rates after 84 h of imbibition. (**D**) Shoot length of germinated seeds after 84 h of imbibition. (**E**) Root length of germinated seeds after 84 h of imbibition. Asterisks indicate significant differences according to Student’s *t*-tests (* *p* < 0.05, ** *p* < 0.01).

The binary vector targeting *GRP* gene was used for rice transformation. A total of 12 positive transgenic plants were obtained in the T_0_ generation. After sequencing the target region in all positive transgenic plants, one transgenic plant with homozygous mutation was obtained. 1-bp deletion (T) occurred at the 3th base from the protospacer adjacent motif (PAM) site (Figure 8A). Thus, the homozygous mutation line was designated as *grp*, whose mature seeds were used for the following germination analysis. In general, germination rate of *grp* mutant was significantly lower than the wild-type control albeit with or without the treatment of BR and GA (Figure 8B,C). Moreover, the lengths of both shoots and roots of the germinated *grp* seeds were also shorter than the control (Figure 8D,E), suggesting that *GRP* mutation has an adverse effect on rice seed germination. Therefore, the identified BR and GA responsive proteins, including GRP, should be involved in BR- and GA-regulated rice seed germination process.

## 3. Discussion

BRs and GAs engage in direct interactions and coordinate a series of plant growth and development events, including seed germination. Nevertheless, it remains unclear how BR and GA coordinate seed germination and what are the common downstream targets that mediate seed germination. In the present study, we identified BR- and GA-responsive proteins in rice embryos during the early stages of seed germination using an iTRAQ proteomic approach and identified downstream targets of BRs and GAs that are intimately involved in the regulation of seed germination. In general, more differentially expressed proteins were identified in mutant *sd1* than that in the BRZ-treated NIP samples. It is possible that GA deficiency caused a more dramatic expression change of downstream regulated proteins, which is consistent with the fact that GA is a major determinant of seed germination. Another possibility is that although BRZ treatment could mimic the BR-deficient conditions, it still could not absolutely exhaust the endogenous BRs. Therefore, a relatively mild effect was caused by pharmaceutical treatment, which might result in a smaller number of changed proteins. A Venn diagram illustrated that a total of 45 common responsive proteins were identified, of which ~64% exhibited coincident changes in expression patterns under BR- or GA-deficient germination conditions, suggesting that crosstalk between BR and GA pathways controls seed germination, at least in part, by modulating the same set of key proteins. Interestingly, GO analysis indicated that most of the common targets were proteins with catalytic activity that participate in various metabolic processes, consistent with the fact that a series of germination events occur in phase II, the most important stage for seed germination, including metabolism reactivation, coleoptile elongation, and mobilisation of reserves [41]. 

Among the common targets, five proteins (glutelin B2, prolamin, glycine-rich protein, pectin esterase, and acyl-desaturase) shared remarkably consistent expression changes (>1.5-fold or <0.67-fold) in both BR- and GA-deficient conditions (Figure S3). For instance, expression of both glutelin B2 and prolamin, two storage proteins in rice seeds, declined significantly when endogenous BR or GA biosynthesis was attenuated, suggesting the decreased accumulation of storage proteins in embryos might contribute partly to the defect in seed germination. This is consistent with previous reports that suppression of seed storage proteins by RNAi, including both glutelins and prolamins, delayed rice seed germination [42]. A recent proteomic study also indicated that the abundance of several glutelin storage proteins was down-regulated during rice seed germination under salt stress [43]. Moreover, abundance of the other three proteins, including glycine-rich protein, pectin esterase and acyl desaturase, was enhanced. Other studies have emphasised a potential correlation between glycine-rich proteins and seed germination. For example, the quantitative trait locus (QTL) *qLTG3-1* controlling low-temperature tolerance at the germination stage was cloned and found to encode a hybrid glycine-rich protein (HyGRP). Strong expression of *qLTG3-1* in embryos enhances rice seed germination under low-temperature conditions by weakening the tissue covering the embryo [44]. To validate our proteomic result and further dissect the function of GRP in seed germination, a *grp* mutant was generated using the clustered regularly interspaced short palindromic repeats-associated protein 9 CRISPR/Cas9 system. Germination analysis showed that *GRP* mutation remarkably suppressed rice seed germination, which could not be fully recovered by GA or BL treatment (Figure 8B–E), suggesting the critical roles of GRP in phytohormone-regulated seed germination. Further diversification analysis of the *HyGRP* gene family in five monocots, namely rice, barley, brachypodium, maize and sorghum, indicated that the *HyGRP* gene family was formed in the ancestral genome before the divergence of these species. The conserved collinearity of chromosomal regions around *qLTG3-1* and its orthologs implies that their functions in seed germination are conserved [45]. In addition, studies indicated that mutation of *PME48*, a member of the pectin esterase family, would cause a significant delay in imbibition and germination of pollen grains in Arabidopsis [46]. On the other hand, enhancing the activity of pectin esterase by direct protein-protein interaction could also improve seed germination performance [47]. However, the function of acyl desaturase in seed germination have not yet been reported yet and require further investigation. 

In addition, several other proteins identified in the present proteomic study also played key roles in seed germination. For example, protein abundance of a plant SKP1-like family protein, component of the SCF (SKP-cullin-F-box protein) E3 ligase complexes that ubiquitinate target proteins, is increased in response to both BR and GA deficiency (Table 2). In *Arabidopsis*, abiotic stress could induce the expression of *Arabidopsis* SKP1-like protein 13 (ASK13); in turn, ASK13 positively regulates seed germination and seedling growth under abiotic stress [48]. Another study also demonstrated that transcript abundance of *GsSKP21* was induced under the treatment of alkali and salt stresses in *Glycine soja*. Overexpression of *GsSKP21* in *Arabidopsis* results in decreased ABA sensitivity and promoted seed germination [49]. Moreover, the accumulation of glutamate decarboxylase (GAD) and alcohol dehydrogenase (ADH) are also promoted in both *sd1* mutant and BRZ-treated Nip (Table 2). GAD functions in catalyzing the unidirectional decarboxylation of glutamate to form γ-aminobutyric acid (GABA). Overexpression of *GAD* could promote the amounts of GABA in Arabidopsis seeds [50]. GABA homeostasis is involved in the regulation of seed germination in both soybean [51] and fava bean [52], and pollen germination in *Picea wilsonii* [53]. As to ADH, its deficient rice mutant *rad* had lower amounts of the α-amylases, resulting in less starch degradation and lower glucose concentrations, subsequent a delayed germination in partially oxygenated water [54]. A rice genotype Khao Hlan On (KHO), which is favorable for direct seedling, accumulates more alcohol dehydrogenase ADH1 and exhibits faster germination than the control under the anaerobic germination condition [55]. Interestingly, the content of Peptidase S10, another identified protein in this study, was decreased in response to both BR and GA deficiency (Table 1). It was reported that Site-2 Protease (S2P), a membrane-associated transcription factor peptidase, desensitizes ABA signaling during seed germination through regulating the expression of negative regulators of ABA signaling, and therefore promotes seed germination [56].

Furthermore, the potential protein association network constructed by STRING analysis highlighted the fact that proteins involved in the metabolic process of seed germination are regulated by BR and GA. For example, alcohol dehydrogenase, with the highest degree of connectivity in the regulatory network, was induced in response to both BR and GA deficiency. A number of reports have suggested that various dehydrogenases are involved in regulating seed germination in sweet sorghum [57], *Arabidopsis* [58] and the legume *Medicago truncatula* [59]. Topoisomerase, another key factor in the centre of the protein association network, can enhance seed germination under salt stress conditions in both *Arabidopsis* [60] and tobacco [61]. The qRT-PCR approach is widely applied to validate proteomic data at the transcriptional level. However, changes in transcript level and protein abundance are not always consistent. Herein, 15 differentially expressed proteins were selected for validation, and only six displayed concordant changes at both transcriptional and translational levels (Figure 7A–D). Such inconsistencies are often not unusual. For instance, transcriptome and proteome analyses were integrated to study the potential mechanism underlying Ogura cytoplasmic male sterility (OguCMS) in cabbage [62]. Using the same set of anther material for RNA-seq and iTRAQ analysis, 1323 differentially expressed genes and 833 differentially abundant proteins were identified. However, only 92 targets overlapped in the transcriptomic and proteomic data, and even more strikingly, only 22 out of the 92 targets shared consistent expression patterns. Similar results have also been reported for both plants and animals, implying a weak correlation between gene transcription and protein abundance [63,64,65,66,67]. Such discrepancies in transcriptomic and proteomic results may be due to the different regulatory mechanisms for mRNAs and proteins. Moreover, genes with distinct functions can be controlled by direct or delayed translation modules, hence delayed translation may also partly contribute to these weak correlations.

## 4. Materials and Methods

### 4.1. Plant Materials

In this study, two different rice materials were used for proteomic experiment; *japonica* cultivar Nipponbare (Nip) that has a functional *SD1* (*SEMIDWARF1*) gene (Nip (*SD1*)), and a near-isogenic line (NIL) of *SD1* locus generated by introduction of a mutated *sd1* allele from *indica* cultivar 9311 into the Nip genetic background, designated Nip (*sd1*). *SD1*, the gene of the Green Revolution, also known as *OsGA20ox2*, encodes a key gibberellin biosynthesis enzyme. *Sd1*, the null allele of *SD1*, produces a semi-dwarf phenotype and was selected during the rice Green Revolution [68,69]. In addition, another *japonica* cultivar Zhonghua 11 (ZH11) was used for *Agrobacterium*-mediated transformation. 

### 4.2. Plant Growth and Seed Germination Assay

Both Nip (*SD1*) and Nip (*sd1*) were grown in the same paddy field at the Wenhui Road Campus, Yangzhou University under identical climatic conditions, and mature seeds were collected for germination assays. Since the seeds of BR-deficient plants were abnormal, and homozygous mutants are often sterile [70], we used brassinazole (BRZ; a commonly used inhibitor of BR biosynthesis) to treat Nip seeds to mimic the BR-deficient germination conditions. Because BRZ was dissolved in dimethyl sulphoxide (DMSO), Nip seeds were imbibed in Milli-Q water supplemented with BRZ or DMSO solution in darkness, while seeds of Nip (*sd1*) were also imbibed in DMSO mock solution. In brief, 100 rice seeds were manually dehulled, sterilised with 70% ethanol, and rinsed twice with sterile water. Seeds were then imbibed with 1 µM BRZor mock solutions at 26 °C in darkness. Embryos of seeds germinated for 36 h were separated and quickly frozen in liquid nitrogen for proteomic analysis. The lengths of shoots and roots of 30 germinated seeds were measured after germination for 60, 72, 84 and 96 h. As to the seed germination assay of *grp* mutant, the seeds were imbibed in Milli-Q water supplemented with 1 µM BL, 10 µM GA or the mock solution, respectively. Three biological replicates were performed for all experiments. 

### 4.3. Protein Extraction

Dithiothreitol (DTT; 10 mM) was added to embryo samples and grinding was performed in liquid nitrogen. The suspension was sonicated for 15 min, centrifuged for 15 min (30,000× *g*, 4 °C), and the supernatant was mixed thoroughly with a 5× volume of chilled acetone containing 10% (*v*/*v*) trichloroacetic acid (TCA) and incubated at ×20 °C overnight. After centrifugation, the precipitate was washed three times with chilled acetone, air-dried, and dissolved in lysis buffer (7 M urea, 2 M thiourea, 4% NP40, 20 mM Tris-HCl, pH 8.0–8.5). The suspension was sonicated and centrifuged as above, the supernatant was transferred to a new tube, and 10 mM DTT was added and incubated at 56 °C for 1 h. Next, 55 mM iodoacetamide (IAM) was added and incubated for 1 h in darkness, and the supernatant was mixed thoroughly with chilled acetone (2 h, −20 °C). After centrifugation, the pellet was air-dried and dissolved in 500 µL of 0.5 M triethylammonium bicarbonate (TEAB; Applied Biosystems, Milan, Italy), sonicated for 15 min (200 W), and the supernatant was collected for proteomic assays.

### 4.4. iTRAQ Labelling and Liquid Chromatography-Electrospray Ionisation-Tandem Mass Spectroscopy (LC-ESI-MS/MS)

In brief, 100 µg of total proteins was digested with Gold-grade Trypsin, dried, reconstituted, and labelled with 8-plex iTRAQ reagent (Applied Biosystems, Foster City, CA, USA). Labelled peptide mixtures were pooled into 20 fractions, desalted, and vacuum-dried. Each fraction was separated and data were acquired using a TripleTOF 5600 System (AB SCIEX, Concord, ON, Canada). A detailed description of iTRAQ labelling and LC-ESI-MS/MS analysis is described in our previous publication [71].

### 4.5. Proteomic Data Analysis

The collected raw data were processed using Proteome Discoverer 1.2 (PD 1.2, Thermo Scientific, San Jose, CA, USA), and the Mascot search engine was used for protein identification. Only peptides with a 95% confidence interval were regarded as positively identified, at least one unique peptide was required for every confident protein identification, and for quantitation, at least two unique peptides were required. Quantitative protein ratios were weighted and normalised by median ratio in Mascot. Only ratios with a fold change >1.2 and *p*-values < 0.05 were considered differentially abundant. 

### 4.6. Bioinformatic Analysis

Cluster software (version 3.0) was used for hierarchical clustering analysis of protein expression patterns [72], and functional annotation of proteins was conducted using the Blast2GO program to search against the non-redundant protein database (NR; NCBI). Gene ontology (GO) was used for gene function classification and description of genes or gene product attributes. Using the PANTHER database, proteins were analysed with four sets of ontologies: molecular function, biological process, cellular component, and protein class [73]. Search Tool for the Retrieval of Interacting Genes/Proteins (STRING) version 10.0 was then used for interactome analysis of identified proteins [74]. Using various prediction methods, including experiments, co-expression, databases, neighbourhood, co-occurrence and text-mining, information on potential protein–protein interactions was retrieved with a confidence cut-off of 0.4.

### 4.7. Quantitative Real-Time Polymerase Chain Reaction (qRT-PCR) Analysis

Total RNA was isolated from the collected embryos of germinated seeds using an RNeasy Plant Mini Kit (Qiagen, Hilden, Germany), and RNA was digested with DNase I (Qiagen) and reverse-transcribed with a SuperScript first-strand synthesis system (Invitrogen, Van Allen Way Carlsbad, CA, USA). After first-strand cDNA synthesis, quantitative real-time polymerase chain reaction (qRT-PCR) was performed using SYBR Premix Ex Taq (TaKaRa, Dalian, Liaoning, China) and an ABI PRISMTM 7700 sequence detector system (Applied Biosystems). The relative transcript abundance was estimated using the 2^−ΔΔCt^ method, and ubiquitin conjugase (UBC) was selected as the reference gene. Primer information is provided in Table S1 in Supplementary Materials.

### 4.8. Plasmid Construction and Rice Transformation

The target site of glycine-rich protein (GRP) was designed for knock out of the gene using CRISPR/Cas9 system. The sgRNA-Cas9 expressing plasmid was constructed following the method described by Wang et al. (2015) [75]. Subsequently, the construct was transformed into *Agrobacterium tumefaciens* EHA105 and then introduced into primary calli derived from mature seeds of *japonica* rice ZH11 via Agrobacterium-mediated transformation [76]. The target sequence of *GRP* and primers for sequencing are provided in Table S1.

## 5. Conclusions

Understanding the mechanisms of seed germination is beneficial for breeding elite rice varieties with high seed vigor. Our proteome analysis identified a number of common responsive proteins of both BRs and GAs during rice seed germination, most of which were involved in the metabolic process and exhibited concordant changes in response to BR and GA deficiency. GO and STRING analyses revealed the functions and possible roles of these proteins in mediating BR- and GA-regulated seed germination. Further functional analysis of one representative protein GRP demonstrated its critical role in normal seed germination. In brief, our study not only provides critical clues for validating BR–GA crosstalk models during rice seed germination, but also helps to optimise the underlying molecular regulatory networks.

## Figures and Tables

**Table 1:** Proteins down-regulated in embryos of germinated rice seeds in response to both BR and GA deficiency by iTRAQ analysis.

Gene ID	NIP (BRZ/Mock) Fold Change ^a^	sd1/NIP Fold Change ^b^	Protein Score ^c^	Unique Peptide ^d^	Sequence Coverage (%) ^e^	Description
Os01g0652800	0.693	0.568	133	3	8.9	Protein of unknown function DUF231
Os02g0248800	0.208	0.586	1443	3	7.1	Similar to glutelin type-B2 precursor
Os02g0249000	0.406	0.776	804	10	22.7	Glutelin
Os03g0231600	0.799	0.712	241	3	9.4	Similar to branched-chain-amino-acid aminotransferase 3
Os03g0240700	0.826	0.731	133	1	10.1	Similar to Erwinia induced protein 2
Os03g0337900	0.831	0.818	371	5	13.5	Similar to predicted protein
Os03g0812000	0.587	0.703	305	2	6.6	DNA topoisomerase type IIA
Os04g0165700	0.804	0.685	312	3	9.5	Cysteine synthase
Os05g0329100	0.555	0.661	917	3	42.7	Prolamin
Os06g0112200	0.812	0.781	274	3	15.4	Purine and other phosphorylases
Os07g0214300	0.474	0.763	1815	2	28.3	Seed allergenic protein RAG2 precursor
Os07g0529600	0.65	0.71	333	6	25.1	Similar to thiazole biosynthetic enzyme 1-1
Os08g0530400	0.465	0.75	71	2	8.8	Moco-containing protein
Os09g0484200	0.808	0.615	2271	7	58	Hypothetical protein
Os11g0213600	0.682	0.785	180	5	11.6	Peptidase S10

^a^ NIP (BRZ/Mock) fold change indicates fold change of protein abundance between BRZ treated samples and the mock-treated control. ^b^ sd1/NIP fold change indicates fold change of protein abundance between sd1 mutant and the NIP control. ^c^ Protein score is based on combined mass spectroscopy (MS) and tandem mass spectroscopy (MS/MS) spectra. The proteins that had a statistically significant (*p* < 0.05) protein score of 70 or more were considered successfully identified. ^d^ Unique peptide indicates the number of identified unique peptides for confident protein identification. ^e^ The protein with a sequence coverage larger than 5% was considered as credible.

**Table 2:** Proteins up-regulated in embryos of germinated rice seeds in response to both BR and GA deficiency by iTRAQ analysis.

Gene ID	NIP(BRZ/Mock)	sd1/NIP	Protein	Unique	Sequence	Description
	Fold Change ^a^	Fold Change ^b^	Score ^c^	Peptide ^d^	Coverage (%) ^e^	
Os01g0358400	1.259	1.229	2745	1	43.4	Similar to 40S ribosomal protein S4
Os01g0880800	1.585	1.506	891	5	16.8	Similar to acyl-[acyl-carrier-protein] desaturase
Os02g0550100	1.38	1.238	419	1	10.8	Similar to vacuolar ATP synthase 16 kDa proteolipid subunit
Os02g0587000	1.529	2.233	569	2	20.8	Similar to glycine-rich protein
Os03g0192400	1.258	1.382	257	3	26.8	GRIM-19 family protein
Os03g0750000	1.288	1.579	2653	1	25.9	Similar to ethylene-responsive protein
Os03g0774200	1.484	1.261	170	4	18.4	Similar to NADH-ubiquinone oxidoreductase subunit 8
Os03g0799000	1.997	1.316	126	2	18.4	Similar to histone H1
Os04g0249600	1.371	1.272	151	2	23.9	Rhodanese-like domain containing protein
Os05g0512600	1.221	1.428	365	2	15.4	X8 domain-containing protein
Os07g0119400	1.554	1.554	142	4	11.6	Similar to pectin esterase-like protein
Os08g0465800	1.304	1.357	952	8	33.4	Similar to glutamate decarboxylase
Os09g0539500	1.367	1.402	784	3	36.6	Similar to SKP1-like protein 1A
Os11g0210500	1.25	1.427	1940	6	39.8	Similar to alcohol dehydrogenase

^a^ NIP (BRZ/Mock) fold change indicates fold change of protein abundance between BRZ treated samples and the mock-treated control. ^b^ sd1/NIP fold change indicates fold change of protein abundance between sd1 mutant and the NIP control. ^c^ Protein score is based on combined mass spectroscopy (MS) and tandem mass spectroscopy (MS/MS) spectra. The proteins that had a statistically significant (*p* < 0.05) protein score of 70 or more were considered successfully identified. ^d^ Unique peptide indicates the number of identified unique peptides for confident protein identification. ^e^ The protein with a sequence coverage larger than 5% was considered as credible.

**Table 3:** Proteins inconsistently regulated in embryos of germinated rice seeds in response to BR and GA deficiency by iTRAQ analysis.

Gene ID	NIP (BRZ/Mock)	sd1/NIP	Protein	Unique	Sequence	Description
	Fold Change ^a^	Fold Change ^b^	Score ^c^	Peptide ^d^	Coverage (%) ^e^	
Os01g0210500	0.762	1.366	1133	6	45	Similar to SOUL-like protein
Os01g0233000	1.237	0.611	391	5	34.3	DREPP plasma membrane polypeptide family protein
Os01g0294700	1.239	0.694	707	8	33.7	Haem peroxidase, plant/fungal/bacterial family protein
Os02g0209300	0.69	1.333	78	1	13.7	Hypothetical conserved gene
Os03g0341100	1.234	0.767	506	4	28.3	Similar to 60S ribosomal protein L18
Os03g0379100	1.252	0.811	176	3	6.9	Protein of unknown function DUF248
Os03g0700400	1.297	0.408	1056	3	37.3	Similar to LOX4
Os03g0842900	0.768	1.28	1047	13	43.9	Similar to steroleosin-B
Os04g0165300	0.825	1.271	74	2	8.9	Conserved hypothetical protein
Os04g0390800	0.818	1.244	2520	16	54.8	NAD(P)-binding domain containing protein
Os04g0497200	1.275	0.735	169	4	9.3	Cellulase precursor
Os04g0546500	0.807	1.31	836	2	13.5	Similar to oleosin
Os05g0268500	0.451	1.478	131	5	12	Similar to serine carboxypeptidase 2
Os05g0474400	1.253	0.775	197	2	10.9	Prenylated rab acceptor PRA1 family protein
Os06g0675700	0.463	1.229	831	4	18	Similar to high pI alpha-glucosidase
Os11g0582400	0.626	1.385	702	2	5.1	Similar to embryo-specific protein

^a^ NIP (BRZ/Mock) fold change indicates fold change of protein abundance between BRZ treated samples and the mock-treated control. ^b^ sd1/NIP fold change indicates fold change of protein abundance between sd1 mutant and the NIP control. ^c^ Protein score is based on combined mass spectroscopy (MS) and tandem mass spectroscopy (MS/MS) spectra. The proteins that had a statistically significant (*p* < 0.05) protein score of 70 or more were considered successfully identified. ^d^ Unique peptide indicates the number of identified unique peptides for confident protein identification. ^e^ The protein with a sequence coverage larger than 5% was considered as credible.

## Supplementary Materials

The following are available online at http://www.mdpi.com/1422-0067/19/11/3460/s1, Figure S1: Alignment of SD1/OsGA20ox2 CDS from Nipponbare (Nip; top) and 9311 (bottom). Four SNPs were detected (shown in red), Figure S2: Genotyping the *sd1* mutant, Figure S3: Five selected responsive proteins displaying consistent prominent changes in protein abundance (fold change ratios ≥1.5 or ≤0.67) in response to both BR and GA deficiency, Table S1: Primers and SG sequence used in this study.
