# Supplementary material for: iTRAQ-Based Analysis of Proteins Co-Regulated by Brassinosteroids and Gibberellins in Rice Embryos during Seed Germination
Source: Int J Mol Sci. 2018 Nov 4;19(11):3460. doi: 10.3390/ijms19113460 (PMC6274883; doi:10.3390/ijms19113460)
Supplement: Supplementary file 1 [file ijms-19-03460-s001.pdf]

# Supplementary data

```
1    ATGGTGGCCGAGCACCCCACGCCACCACAGCCGCACCAACCACCGCCCATGGACTCCACC
     ||||||||||||||||||||||||||||||||||||||||||||||||||||||||||||
1    ATGGTGGCCGAGCACCCCACGCCACCACAGCCGCACCAACCACCGCCCATGGACTCCACC

61   GCCGGCTCTGGCATTGCCGCCCCGGCGGCGGCGGCGGTGTGCGACCTGAGGATGGAGCCC
     ||||||||||||||||||||||||||||||||||||||||||||||||||||||||||||
61   GCCGGCTCTGGCATTGCCGCCCCGGCGGCGGCGGCGGTGTGCGACCTGAGGATGGAGCCC

121  AAGATCCCGGAGCCATTCGTGTGGCCGAACGGCGACGCGAGGCCGGCGTCGGCGGCGGAG
     ||||||||||||||||||||||||||||||||||||||||||||||||||||||||||||
121  AAGATCCCGGAGCCATTCGTGTGGCCGAACGGCGACGCGAGGCCGGCGTCGGCGGCGGAG

181  CTGGACATGCCCGTGGTCGACGTGGGCGTGCTCCGCGACGGCGACGCCGAGGGGCTGCGC
     ||||||||||||||||||||||||||||||||||||||||||||||||||||||||||||
181  CTGGACATGCCCGTGGTCGACGTGGGCGTGCTCCGCGACGGCGACGCCGAGGGGCTGCGC
                                                              Glu
241  CGCGCCGCGGCGCAGGTGGCCGCCGCGTGCGCCACGCACGGGTTCTTCCAGGTGTCCGAG
     |||||||||||||||||||||||||||||||||||||||||||||||||||||||||| |
241  CGCGCCGCGGCGCAGGTGGCCGCCGCGTGCGCCACGCACGGGTTCTTCCAGGTGTCCGGG
                                                              Gly
301  CACGGCGTCGACGCCGCTCTGGCGCGCGCCGCGCTCGACGGCGCCAGCGACTTCTTCCGC
     ||||||||||||||||||||||||||||||||||||||||||||||||||||||||||||
301  CACGGCGTCGACGCCGCTCTGGCGCGCGCCGCGCTCGACGGCGCCAGCGACTTCTTCCGC

361  CTCCCGCTCGCCGAGAAGCGCCGCGCGCGCCGCGTCCCGGGCACCGTGTCCGGCTACACC
     ||||||||||||||||||||||||||||||||||||||||||||||||||||||||||||
361  CTCCCGCTCGCCGAGAAGCGCCGCGCGCGCCGCGTCCCGGGCACCGTGTCCGGCTACACC

421  AGCGCCCACGCCGACCGCTTCGCCTCCAAGCTCCCATGGAAGGAGACCCTCTCCTTCGGC
     ||||||||||||||||||||||||||||||||||||||||||||||||||||||||||||
421  AGCGCCCACGCCGACCGCTTCGCCTCCAAGCTCCCATGGAAGGAGACCCTCTCCTTCGGC

481  TTCCACGACCGCGCCGCCGCCCCCGTCGTCGCCGACTACTTCTCCAGCACCCTCGGCCCC
     ||||||||||||||||||||||||||||||||||||||||||||||||||||||||||||
481  TTCCACGACCGCGCCGCCGCCCCCGTCGTCGCCGACTACTTCTCCAGCACCCTCGGCCCC

541  GACTTCGCGCCAATGGGGAGGGTGTACCAGAAGTACTGCGAGGAGATGAAGGAGCTGTCG
     ||||||||||||||||||||||||||||||||||||||||||||||||||||||||||||
541  GACTTCGCGCCAATGGGGAGGGTGTACCAGAAGTACTGCGAGGAGATGAAGGAGCTGTCG
```

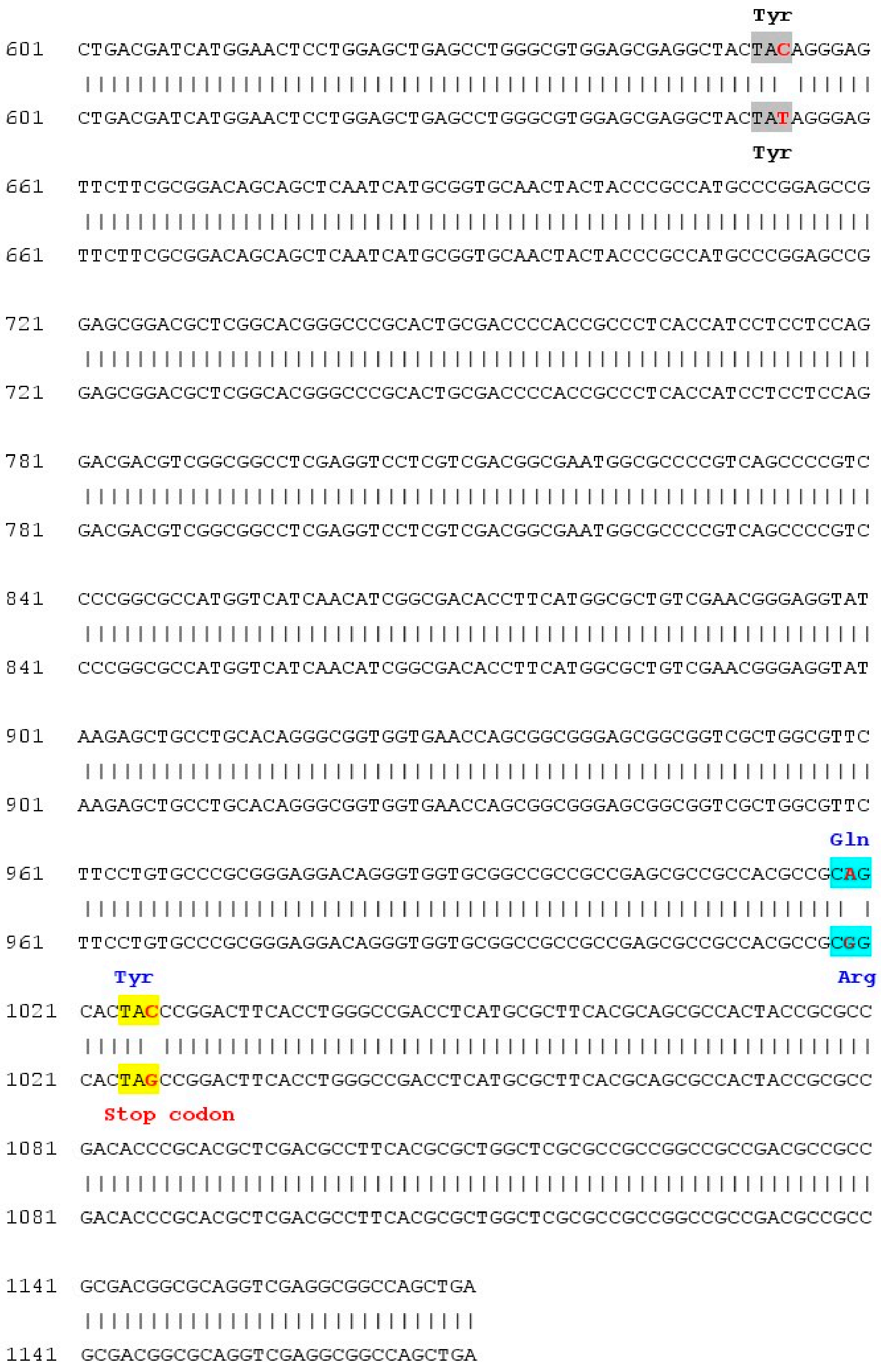


Figure S1. Alignment of *SD1/OsGA20ox2* CDS from Nipponbare (Nip; top) and 9311 (bottom). Four SNPs were detected (shown in red). The genetic codon associated with each SNP is labelled by a coloured box as follows: grey = no change in the encoded

amino acid (aa); light blue = a change in aa; yellow = a change from an aa to a stop codon.

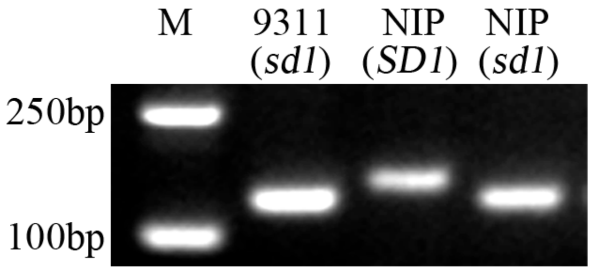


Figure S2. Genotyping the *sd1* mutant. A pair of primers (SD1-F and SD1-R) were used to differentiate Nip-type *SD1* and the 9311-type mutated *sd1* allele.

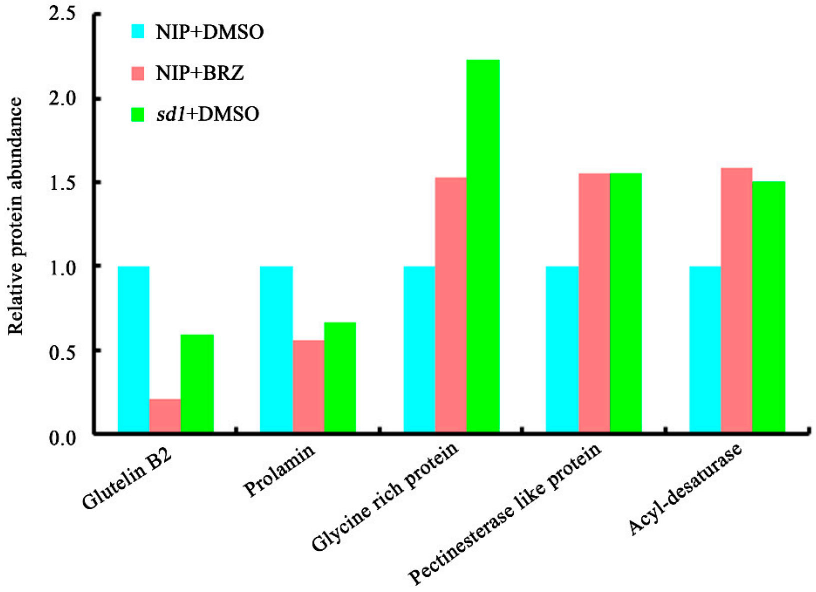


Figure S3. Five selected target proteins displaying consistent prominent changes in protein abundance (fold change ratios ≥1.5 or ≤0.67) in response to both BR and GA deficiency.

**Table S1.** Primers and SG sequence used in this study

| Primer Names | Sequence ( 5' → 3' ) | Usage |
|---|---|---|
| Os02g0587000-SG-seq | GAACGCGATGAGAGCCTGCC | SG sequence |
| Os02g0587000seq-F | GGACTATGAGCAAGCGTCGT, | Primers for |
| Os02g0587000seq-R | CACCTTGGTGTCCTCCTTCT | sequencing |
| Os02g0587000qRT-F | GCGGCGCGTATGATCTCTAT | |
| Os02g0587000qRT-R | ACATCGGTCACACACACAGT | |
| Os03g0774200qRT-F | CCTACATGGGCGCAGGGAA | |
| Os03g0774200qRT-R | TTGAGTGTCAGCATCAAACCG | |
| Os03g0799000qRT-F | CGAGCGAGCAATCTAGGGC | |
| Os03g0799000qRT-R | CCCCTCCTCGCAACTAACAA | |
| Os07g0119400qRT-F | AGCGAGGCATACAGTTCAGG | |
| Os07g0119400qRT-R | TACCGCAGAGTATGGCGTTG | |
| Os02g0249000qRT-F | CAAAGACAGAGCGACCAAGC | |
| Os02g0249000qRT-R | TTGCCCTCTCTGGTTGTTACC | |
| Os03g0240700qRT-F | GGAAGAACGAGCACAGGTTG | Primers used |
| Os03g0240700qRT-R | GAGGCGTTTCTCAGTCTCCT | for qRT-PCR |
| Os03g0812000qRT-F | CCTGAATGCACAAGAGCTGC | analysis |
| Os03g0812000qRT-R | TCATCATCCCCACAACGACG | |
| Os01g0880800qRT-F | AGACCGTCCAGTACCTCATCG | |
| Os01g0880800qRT-R | GGCATGGTGATCCGCTTGTG | |
| Os02g0209300qRT-F | GATCAATCGGCTCAGGGAGG | |
| Os02g0209300qRT-R | GAAGTGCAGGTTGGTAGGGG | |
| Os04g0390800qRT-F | TCCCTCACCTGAAGAAGACGC | |
| Os04g0390800qRT-R | GGTGATCTCCGACTCGACCA | |
| Os06g0675700qRT-F | CAGGTACACCGCTCACTGG | |
| Os06g0675700qRT-R | GAGTGATCCCTCGAGAACGG | |
| Os011g0582400qRT-F | GAGAGAGAGCAACACTGCCG | |
| Os011g0582400qRT-R | TTACACTGCCTGGAGGATGG | |
| Os01g0233000qRT-F | GACCAAGGTTCTCACTGGAC | |
| Os01g0233000qRT-R | CGGGGAAATCAATCTTAGCCA | |
| Os03g0379100qRT-F | CGCCTGATTTCTCCTCCCCG | |
| Os03g0379100qRT-R | GCTATGCTGTCTCCTCTTCCG | |
| Os03g0700400qRT-F | CTGTCGCTGGTGGAGCAGAT | |
| Os03g0700400qRT-R | CGCCTTGATCGAGTAGCCCA | |
| OsUBCqRT-F | CCGTTTGTAGAGCCATAATTGCA | |
| OsUBCqRT-R | AGGTTGCCTGAGTCACAGTTAAGTG | |
